# Slower Fibrosis Progression Among Liver Transplant Recipients With Sustained Virological Response After Hepatitis C Treatment

**DOI:** 10.14740/gr686w

**Published:** 2015-10-21

**Authors:** Shahid Habib, Edward Meister, Sana Habib, Traci Murakami, Courtney Walker, Abbas Rana, Obaid S. Shaikh

**Affiliations:** a Liver Institute, Department of Internal Medicine, Divisions of Gastroenterology, Hepatology and Transplantation, University of Arizona, AZ, USA; b Department of Medicine, University of Arizona, AZ, USA; c Division of Transplantation Surgery, Department of Surgery, Baylor College of Medicine, TX, USA; d Division of Transplantation Surgery and Thomas E. Starzl Transplantation Institute, University of Pittsburgh School of Medicine, Pittsburgh, PA, USA

**Keywords:** Modified Ishak-Knodell activity index, Retrospective study, Liver allograft, Fibrosis progression, Patient and graft survival

## Abstract

**Background:**

The natural course of hepatic fibrosis in HCV allograft recipients with sustained virological response (SVR) after anti-HCV therapy remains debatable. The aim of this study was to examine the progression of fibrosis in a cohort of patients who achieved SVR compared with those without treatment.

**Methods:**

The 167 patients who met the inclusion and exclusion criteria were chosen from a transplant database. All patients were required to have histological evidence of recurrent HCV infection post-liver transplantation and a follow-up biopsy. The 140 of these patients had received anti-viral therapy. Twenty-seven patients were identified as controls and were matched with the treatment group in all respects. The patients were categorized into four groups based on treatment response: 1) no treatment (control) (n = 27); 2) non-responders (n = 81); 3) relapsers (n = 32); and 4) SVR (n = 27). The endpoint was the stage of fibrosis on the follow-up liver biopsy.

**Results:**

The treated and untreated groups were similar in clinical characteristics at the time of transplantation and prior to the initiation of treatment. The 72% of the cohort showed a fibrosis progression of ≥ 1 stage; this change did not significantly differ between the patient groups. Nonetheless, the fibrosis progression rate was the highest in the untreated group and lowest in the patients who achieved SVR. A coefficient of determination was used. Improvements in fibrosis scores were found with greater treatment duration. These improvements were most evident with the achievement of SVR.

**Conclusions:**

In conclusion, SVR after anti-viral therapy for recurrent hepatitis C infection post-transplantation was associated with slower fibrosis progression and significantly improved graft survival.

## Introduction

Chronic hepatitis C virus (HCV) is the leading indication for liver transplantation in the United States. Graft reinfection with hepatitis C occurs universally after liver transplantation and is a significant factor in graft loss, re-transplantation and increased mortality. In the liver allograft, the course of HCV infection is often aggressive and results in lower rates of graft and patient survival compared with patients without HCV infection [1]. Up to 28% of patients develop cirrhosis within 5 years of transplantation [2]. Additionally, severe cholestatic hepatitis develops in approximately 10% of HCV-infected graft recipients, usually within 6 months of transplantation, and results in graft loss if left untreated [3, 4]. Treatment efficacy with standard or pegylated interferon and ribavirin is also suboptimal compared with immunocompetent individuals [5, 6], and the efficacy of direct antiviral agents has not been well studied in liver allograft recipients. However, preliminary results are very promising [7, 8].

In pre-transplant HCV cirrhotic patients, achieving sustained viral response (SVR) after anti-HCV treatment has clearly resulted in fibrosis regression [9-11]. However, the effect of SVR on fibrosis progression is not well studied in post-liver transplantation HCV patients. We performed PubMed (MeSH) and other data searches and identified 10 studies that investigated the effects of SVR on fibrosis progression [12-21]. Eight out of 10 studies are from Europe, and two are from the USA. Not all of the studies were designed to investigate changes in fibrosis, which was analyzed in a subgroup of the study cohort. All studies showed improvements in necroinflammatory score; however, fibrosis improvement was not universal, and there was variability in how the fibrosis data was reported [12-21] (Table 1).

**Table 1:** Literature Review [12-21]

Author	Year	Country	N	Study design	CG	FU	Pre-treatment fibrosis	Effect on fibrosis: SVR	Effect on fibrosis: NR	Effect on fibrosis: control	P value
Tame et al [15]	2013	Italy	35*	Prospective	N	40	Metavir 2/4	(n = 9) 32% improved, 68% unchanged, 0% worsened	(n = 19) 38% improved, 66% unchanged, 16% worsened	(n = 7) 70% improved, 50% unchanged, 50% worsened	0.05
Belli et al [12]	2012	Italy	69*	RCT	Y	36	Ishaq-Knodell ≤ 3/6	(n = 15) 20% worsening	(n = 21) 47.6% worsening	(n = 36) 50% worsening	0.04
Carrion et al [16]	2007	Spain	81	RCT	Y	6	Metavir 2/4	(n = 18) 50% improved, 50% stable, 0% worse	(n = 36) 6% improved, 36% stable, 58% worse	(n = 27) 4% improved, 26% stable, 70% worse	0.009
Bizollon et al [13]	2007	France	25*	Prospective	Y	6	Unknown	(n = 8) 100% improved, 0% stable, 0% worse	(n = 17) 65% improved, 35% stable, 0% worse	(n = 21) 5% improved, 19% stable, 76% worse	Significant; P value not presented
Oton et al [17]	2006	Spain	15*	Retrospective	N	6	Ishaq-Knodell ≥ 1/6	(n = 7) mean score pre-treatment: 2.4/6, mean score post-treatment: 2.6/6	(n = 8) mean score pre-treatment: 2.7/6, mean score post-treatment: 3.7/6		NS
Fernandez et al [18]	2006	Spain	16*	Prospective	N	6	Scheuer Fibrosis score	(n = 7) mean score pre-treatment: 1.5/4, mean score post-treatment: 1.1/4	(n = 9) mean score pre-treatment: 2.4/6, mean score post-treatment: 2.8/6		NS
Barenguer et al [19]	2009	Spain	47*	Retrospective	N	12	HAI fibrosis stage 0 - 4	No change and improvement 46%	No change and improvement 44%		NS
Stravitz et al [21]	2004	USA	23	Retrospective	N	23		(n = 11) mean score pre-treatment: 1.9/4, mean score post-treatment: 1.5/4	(n = 12) mean score pre-treatment: 2.5/4, mean score post-treatment: 2.7/4		NS
Abdelmalek et al [20]	2004	USA	26*	Retrospective	N	24 - 60	Not known	At year 2 FU, 27% improved, 38% unchanged, 35% worsened, At year 3-5 FU, 67% improved, 13% unchanged, 20% worsened.			0.05
Bahra et al [14]	2007	Germany	28	Prospective	N	36	1.8	(n = 28) 18% improved, 60% unchanged, 21% worsened. Mean fibrosis stage at 1, 3 and 5 years: 2.0, 2.1 and 1.4 respectively.			NS

N: number of patients; NS: not significant; *: number of patients evaluated histologically; CG: control group; NR: non-responders; SVR: sustained virological response; FU: follow-up.

One study found that the grade of inflammation and fibrosis stage improved from baseline histology in the majority of HCV patients treated with interferon and ribavirin post-transplantation [16]. Another study found that interferon treatment of recurrent hepatitis C did not consistently improve histology after virological response and may even increase the risk of allograft rejection [17]. There is significant heterogeneity between studies, and only a minority of patients undergo liver biopsy both prior to and after antiviral therapy. Patients who achieved SVR post-transplantation have graft and patient survival rates similar to those without HCV infection [22-27]. Nevertheless, patients who remain virological are at risk for progressive disease [22-24]. In summary, there is no conclusive evidence indicating that achieving SVR with anti-HCV therapy post-transplantation results in fibrosis regression and the question remains debated.

To clarify whether treating hepatitis C results in any level of fibrosis regression, we examined the effect of antiviral therapy for HCV on the progression of fibrosis in a large cohort of patients who received liver transplants for hepatitis C cirrhosis. We compared patients who received antiviral therapy (an interferon-based regimen that included protease inhibitors) with those who were not treated (control). Liver fibrosis progression rates were analyzed in relation to virus clearance post-treatment. To our knowledge, this is the largest cohort studied in this regard.

## Materials and Methods

### Study population

The protocol was approved by the institutional review board at the Thomas E. Starzl Transplantation Institute at the University of Pittsburg. Adult patients aged 18 years and older who were transplanted for HCV cirrhosis were included for retrospective analysis. The patients were identified using an electronic database that prospectively enrolled liver transplant candidates and recipients. The diagnosis of hepatitis C was based on a positive anti-HCV test and HCV RNA. All patients were required to have histological evidence of recurrence prior to the initiation of therapy (baseline biopsy) and have had a follow-up biopsy at least 6 months post-treatment.

### Anti-HCV therapy

Patients who were identified to have abnormal aminotransferases or cholestatic hepatitis were suspected of having recurrent HCV infection after the exclusion of other etiologies. All patients were considered for treatment once histologic recurrence was established. Anti-HCV therapy included unmodified interferon-α2b (INTRON^®^ A), pegylated interferon-α2b (Peg-Intron^®^) and pegylated interferon-α2a (Pegasys^®^), ribavirin and protease inhibitors (telaprevir and boceprevir). Treatment length varied according to side effects, tolerance and response to treatment. Genotype 2 and 3 patients received treatment for 6 months. In patients with other genotypes, treatment was planned for at least 48 weeks. However, in some patients, treatment was discontinued prematurely because of significant side effects and/or no response to treatment. In others, treatment was prolonged per response-guided therapy. The patients were considered to have SVR if HCV RNA was negative at 6 months after the completion of treatment. The treatment group was categorized based on response to treatment: SVR, relapsers and non-responders.

### Control group

The control group comprised patients who were determined to have histological recurrent HCV infection and were considered for treatment but refused to undergo treatment. They also have had follow-up biopsies. These patients were matched with the treatment group in all respects.

### Histological assessment

All allograft biopsies were included in the analysis. The biopsies were read routinely by one of the liver pathologists at our institution and subsequently reviewed by another pathologist. The severity of inflammation and fibrosis was graded and staged according to the modified Ishak-Knodell activity index (MHAI), and the severity of allograft rejection was scored according to the Banff schema [28, 29].

### Study endpoints

The primary endpoint of the study was fibrosis progression rate (stage of fibrosis on the last follow-up liver biopsy) in the hepatic allograft.

### Statistical analysis

Chi-square and ANOVA one-way analysis were used to compare baseline clinical features for the dichotomous and continuous variables, respectively. A Kaplan-Meier analysis with a log-rank test was performed for survival estimates. The log delta time was calculated to minimize the effect of variable duration between the two biopsies. An R-squared regression analysis was used to measure the percentage of the total variation in the change of fibrosis as explained by the regression model. A non-linear cubic fit model was used to display a comparison between the untreated groups vs. the treatment groups in terms of the achievement of SVR. As the R-squared value increases, the observed values are more closely fit to the regression model. This would indicate a favorable and more impressive regression score. A P value of ≤ 0.05 was considered to be statistically significant. A statistical software package was used (SPSS 22 for Windows 2008; SPSS Inc., Chicago, IL, USA).

## Results

### Study cohort transplantation course

A total of 167 patients met the inclusion and exclusion criteria and underwent the final analysis. The study cohort was categorized into two main groups: a no treatment group (n = 27) and a treatment group (n = 140). The treatment group consisted of three subgroups: 1) non-responders (n = 81); 2) responder relapsers (n = 32); and 3) SVR (n = 27). The recipient, donor and transplant variables of the overall study cohort were evaluated (Table 1). The majority of transplant recipients were males (78%) with a mean age of 49 years, a mean BMI of 28.30 and the majority of recipients were white (90%). Of the participants, 96% were non-Hispanic. The 2% of the recipients were older than 65 years.

The demographic, recipient, donor and immunosuppression features were similar between the control and treatment groups (Table 2). At the time of transplantation, the severity of liver disease as indicated by the model for end-stage liver disease (MELD) scores and Child-Pugh scores were similar between the two groups. Both groups were also similar regarding post-transplant course and immunosuppression management. The majority of patients received tacrolimus-based immunosuppressive regimens, and some received induction immunosuppression with either anti-thymocyte globulin (Thymoglobulin^®^) or alemtuzumab (Campath^®^). The use of either intravenous or oral corticosteroids was similar between the two groups. Steroid bolus use for acute rejection episodes was also similar in both groups.

**Table 2:** Clinical Characteristics of Patients Prior to Anti-HCV Therapy

Features	Study cohort (n = 167)	Control group (n = 27)	Treatment group (n = 140)	P value
Patient age, mean ± SD	49.4 ± 7.7	48.6 ± 9.09	49.5 ± 7.4	NS
Patient ≥ 65, % (n)	2.4 (4)	3.7 (1)	2.1 (3)	NS
Gender male, % (n)	78.4 (131)	74.1 (20)	79.3 (111)	NS
Race white, % (n)	89.8 (150)	88.9 (15)	90 (126)	NS
Non-Hispanic, %	95.8 (160)	88.9 (24)	90 (126)	NS
Donor age, mean ± SD	40.6 ± 17.3	41.1 ± 18.8	40.5 ± 17.1	NS
Donor age, % > 50	31.7 (53)	29.6 (8)	32.1 (45)	NS
Donor gender, % male	55.7 (53)	55.6 (15)	55.7 (78)	NS
Donor ethnic, % white	85 (142)	81.5 (22)	85.7 (120)	NS
Donor ethnic, % non-Hispanic	83.8 (140)	77.8 (21)	85 (119)	NS
Donor NHBD, % (n)	6.3 (4)	00(00)	7.7 (4)	NS
MELD score, mean ± SD	16.4 ± 7.1	18.2 ± 7.9	16 ± 6.9	NS
Child-Pugh score, mean ± SD	7.8 ± 1.58	7.9 ± 1.5	7.8 ± 1.5	NS
Cold ischemia time, h ± SD	11.5 ± 3.5	12.9 ± 3.9	11.3 ± 3.4	NS
Warm ischemia time, min ± SD	34.8 ± 21.1	28.5 ± 24.0	36 ± 20.4	NS
Induction therapy, % (n)	16.8 (28)	14.8 (4)	17.1 (24)	NS
Received steroids, % (n)	81.6 (129)	87 (20)	80.7 (109)	NS

n: number of patients; NHBD: non-heart beating donor; MELD: model for end-stage liver disease; CTP: Child-Turcotte-Pugh.

### Anti-HCV treatment course

All patients developed recurrent HCV infection within 6 months of transplantation. They manifested with abnormal aminotransferases, and the mean aspartate transaminase (AST) and alanine aminotransferase (ALT) were 91 and 100 IU/mL, respectively. The majority of patients were non-cholestatic with a mean bilirubin of 1.2 mg/dL and alkaline phosphatase of 190 IU/mL. Pre-treatment biopsy was performed at a mean interval of 335 days (11 months) with SD ± 410 days from the date of transplantation. Histopathology showed an Ishak-Knodell MHAI grade of 4.9/18 with SD ± 2.6 and fibrosis stage of 1.3/6 with SD ± 1.4. Treatment was initiated at a mean interval of 375 days (12.3 months) with an SD of ± 427 days. The mean bilirubin and creatinine at initiation of treatment were 1.8 mg/dL and 1.2 mg/dL, respectively. Treatments continued per the guidelines: 24 weeks for genotype 2 and 3 and ≥ 48 weeks for genotype 1. Treatments were prematurely discontinued for adverse events. Some patients received response-guided therapy, especially with protease inhibitors. The control group was carefully selected to match the treatment group regarding pre-treatment characteristics such as general features, biochemistry, time of biopsy and histopathology (Table 3). The majority of the treatment group (69%) received treatment for ≥ 48 weeks, and 21% received 24 - 47 weeks of treatment. A minority of patients received treatments for up to 24 weeks. The rapid virological response and early virological response could not be precisely determined due to the retrospective nature of the study; however, 42% of the treatment group responded to the therapy by achieving an HCV-negative status during treatment. The 16% of the treatment group was confirmed to have SVR status. The mean duration of the follow-up biopsy was 1.5 years after the completion of treatment. The mean interval between the two biopsies was 2.9 years.

**Table 3:** Anti-HCV Treatment Course

Clinical features	Control (n = 27)		Treatment (n = 140)		P value
	Mean	SD	Mean	SD	
Bilirubin pre-treatment, mg/dL	0.91	0.61	1.29	1.92	NS
Bilirubin at initiation, mg/dL	2.86	6.07	1.61	3.47	NS
Bilirubin at follow-up, mg/dL	5.49	9.14	3.77	8.53	NS
ALT pre-treatment, IU/dL	121.56	122.84	96.89	103.40	NS
AST pre-treatment, IU/dL	99.11	115.61	90.09	130.42	NS
AST/ALT pre-treatment	0.89	0.51	0.92	0.42	NS
ALP pre-treatment, IU/dL	275.73	311.36	171.11	190.02	NS
Cholestasis index	2.29	2.46	2.21	1.95	NS
Creatinine pre-treatment, mg/dL	1.35	0.51	1.26	0.42	NS
Time from transplant to pre-treatment biopsy, years	0.99	1.46	0.90	1.05	NS
MHAI grade 0 - 18 pre-treatment	5.20	1.86	4.93	2.78	NS
Fibrosis stage 0 - 6 pre-treatment	1.11	1.31	1.34	1.46	NS
Time from transplant to treatment, years			0.98	1.06	NS
Treatment duration, years			1.62	1.16	NS
Time from transplant to follow-up biopsy, years	3.29	2.31	3.97	2.89	NS

SD: standard deviation; ALT; alanine aminotransferase; AST: aspartate aminotransferase; ALP: alkaline phosphatase; MHAI: modified histologic activity index.

### Fibrosis analysis

**Figure 1:**
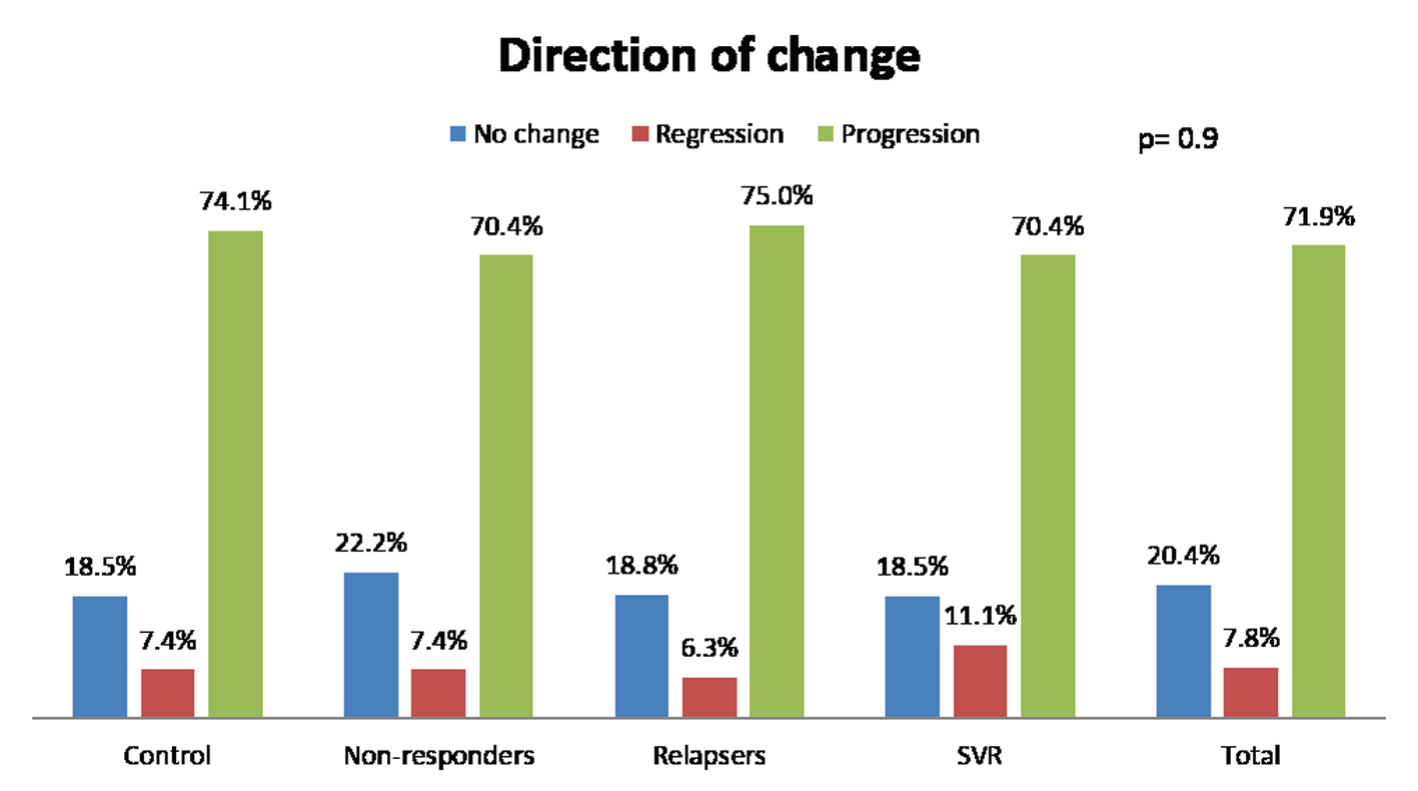
Change of fibrosis in patients. SVR: sustained virological response.

**Figure 2:**
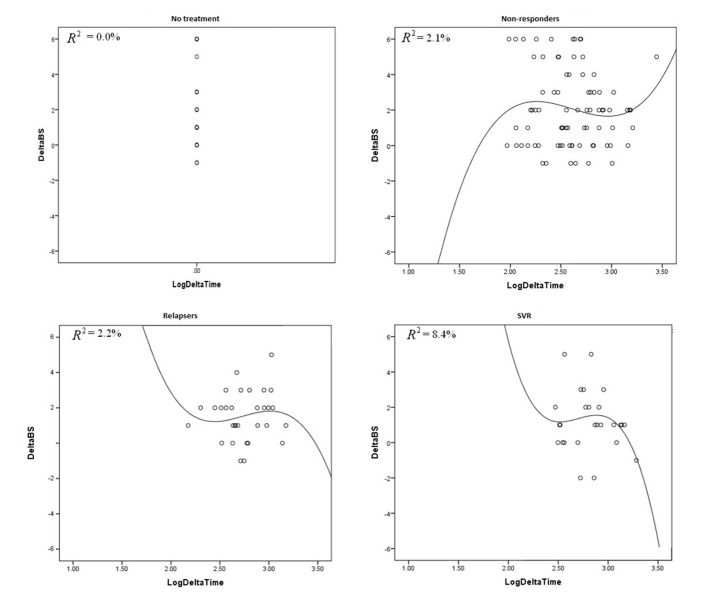
These graphs plot R^2^ against delta log time in all treatment groups and control group.

**Figure 3:**
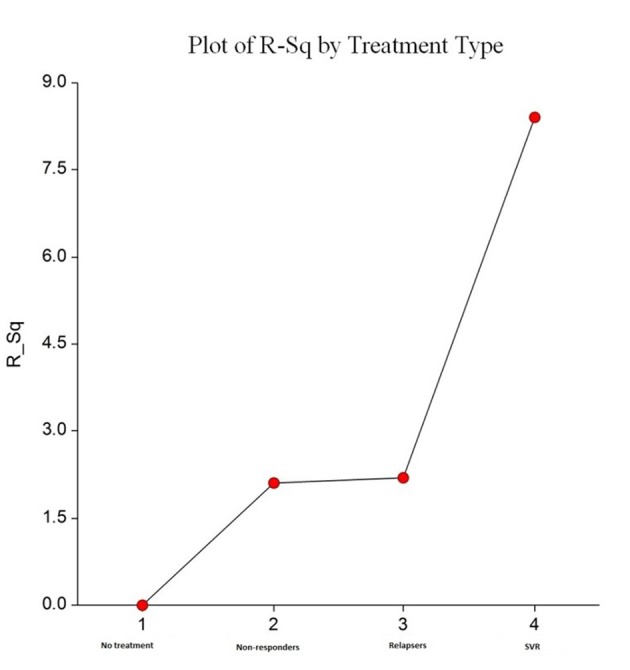
Plots R^2^ by treatment type. This shows increasing trend in percentage of R^2^ for a non-linear cubic model fit from “no treatment” to “SVR treatment”. This indicates favorable improvement in time dependent fibrosis scores, and this was most evident with SVR.

We analyzed fibrosis progression in the three treatment response subgroups (SVR, relapsers and non-responders) and compared them with the control group. The control group was similar to the treatment groups regarding the timing of biopsies and pre-treatment histologic disease activity (Table 4). There was ≥ 1 stage progression of fibrosis in 72% of the study cohort with a similar progression in each group without achieving statistical significance (Fig. 1). The mean change in fibrosis score among all treatment subgroups and the control group was statistically similar; however, there was a trend toward a smaller magnitude of change in the control group to SVR (P = 0.08). Potential correlations regarding the time interval between the biopsies and the change in fibrosis score (time-dependent fibrosis change) were assessed in all study groups. The fibrosis progression rate in all four groups was highest in the control group (1.02 stages per year) and lowest in patients who achieved SVR (0.45 stages per year); nevertheless, there was a trend toward slower progression in patients with SVR (P = 0.08). Using a non-linear cubic model, there was an improvement in fibrosis scores that was most strongly demonstrated in the patients who achieved SVR as opposed to the patients who achieved relapse or have not responded (Fig. 2). Overall, a trend of slower progression of fibrosis over time was observed in patients who achieved SVR (Fig. 3).

**Table 4:** Fibrosis Analysis

	Control group	Non-responders	Relapsers	SVR	P value
Time interval between LT and initiation of treatment, years		0.89	1.17	1.01	NS
Time interval between LT and first liver biopsy, years	1.03	0.81	1.06	0.96	NS
Time interval between LT and second liver biopsy, years	3.40	4.12	3.47	2.92	NS
Time interval between first and second liver biopsy, years	2.37	3.32	2.40	3.00	Ns
Mean fibrosis change between two biopsies	2.04	2.11	1.57	1.37	0.08
Mean fibrosis stage change per year	1.02	0.63	0.65	0.45	0.08
Direction of change in fibrosis					NS
Regression, % (n)	7.4 (2)	7.4 (6)	6.2 (2)	11.1 (3)	
No change, % (n)	18.5 (5)	22.2 (18)	18.8 (6)	18.5 (5)	
Progression, % (n)	74.1 (20)	70.4 (57)	75 (24)	70.4 (19)	

LT: liver transplantation.

### Survival analysis

**Figure 4:**
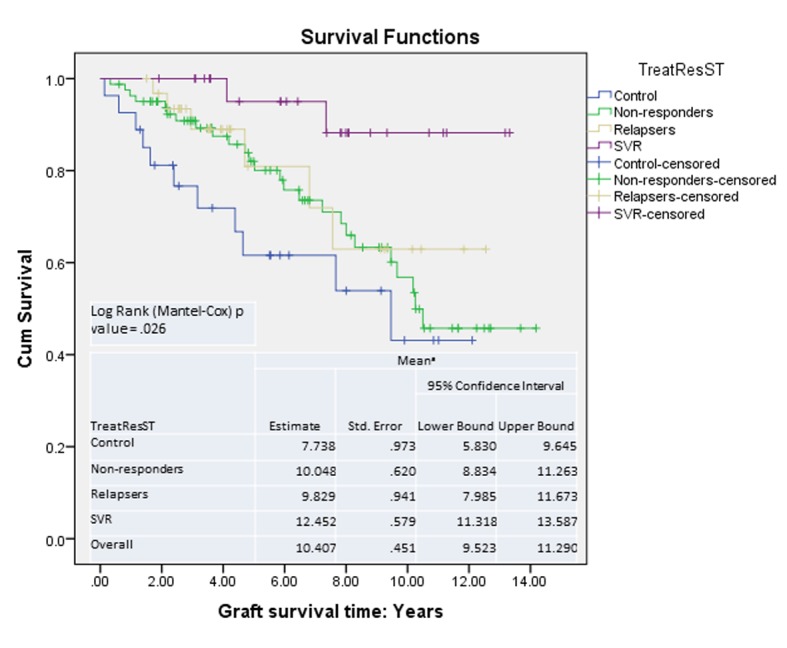
Graft survival: Kaplan-Meier survival curves.

Graft survival analysis showed improved survival in patients with SVR compared with those without (Fig. 4). The risk of mortality was higher if the patient remained untreated or did not respond to treatment (HR: control 7.6 (P = 0.008), NR 4.7 (P = 0.034), relapsers 3.7 (P = 1.05)).

### Conclusions

The rigors of a well-designed, controlled study are difficult to apply to transplanted patients primarily due to the concurrent hematologic and renal side effects of immunosuppression. We initially designed a prospective clinical trial to study anti-HCV therapy in liver transplant recipients; this prospective trial was ended because of enrollment failure [30]. Several uncontrolled studies have examined the efficacy of unmodified interferon and ribavirin monotherapies, as well as that of an interferon and ribavirin combination in patients with histological recurrent HCV infection [5, 31-40]. It remains unknown whether achieving SVR with anti-HCV treatment can stabilize fibrosis progression.

We performed a data search and analyzed the results of all of the studies evaluating the effect of SVR on hepatic fibrosis (Table 1). Among them, two were randomized control trials [12, 16], and both showed a significant regression of fibrosis over a period of 6 months to 2 years following the completion of treatment; however, one study did not differentiate between stable and improved fibrosis. Other studies from Europe evaluated only a small number of patients showing similar patterns of fibrosis progression in SVR, non-responders and control groups. Two case control studies showed improvements in histology; however, these were not compared with non-responders or control groups [14, 20].

Our study evaluated a large cohort of post-transplant HCV patients and did not demonstrate significant histologic regression in patients who achieved SVR with anti-HCV therapy compared with other groups (including controls). Treating recurrent hepatitis C in liver transplant recipients appears to be beneficial because antiviral therapy is associated with a slower progression of fibrosis with SVR status. Our study clearly confirmed improved graft survival, as previously published. Our study did not confirm fibrosis regression, as was shown in two previously published clinical studies [12, 16]. Our results are consistent with those of other studies and confirmed slower progression with SVR status. Clinically, this finding is important and stresses the need for prompt treatment. All patients should be offered anti-HCV treatment, especially in the era of direct-acting antivirals (DAAs).

Significant evidence indicates that SVR status results in fibrosis regression in a pre-transplant setting but in slower fibrosis progression in a post-transplant setting. The underlying mechanism of this differing behavior is unknown. Some evidence indicates that achieving SVR resulted in an increased risk of both acute and chronic rejection [21]. Whether this was truly the effect of the interferon remains unknown. Immunosuppressive therapy is well known to cause insulin resistance and is associated with non-alcoholic steatohepatitis (NASH). No studies, including ours, have looked into the features of NASH in follow up biopsies.

The pitfalls of our study are due to its retrospective nature, though the data was collected prospectively from a transplant database. The interval between biopsies was not uniform, and the follow-up interval was relatively short (1.5 years after the completion of treatment).

In conclusion, our study shows that achieving SVR with anti-HCV therapy in liver transplant recipients does not result in fibrosis regression. This is in contrast to some previously published data; however, SVR via anti-HCV therapy slows the progression of fibrosis over time, which translates into significant improvements in graft survival. Thus, all patients with recurrent HCV infection should be considered for treatment at an early recurrence stage.

## Abbreviations

ALT: alanine aminotransferase
AST: aspartate transaminase
DAAs: direct acting antivirals
HCV: hepatitis C virus
LT: liver transplant
MELD: model for end-stage liver disease
MHAI: modified Ishak-Knodell activity index
NHBD: non-heart beating donor
NS: not significant
SD: standard deviation
SVR: sustained viral response
